# Immune Defense in Upper Airways: A Single-Cell Study of Pathogen-Specific Plasmablasts and Their Migratory Potentials in Acute Sinusitis and Tonsillitis

**DOI:** 10.1371/journal.pone.0154594

**Published:** 2016-04-29

**Authors:** Nina V. Palkola, Karin Blomgren, Sari H. Pakkanen, Ritvaleena Puohiniemi, Jussi M. Kantele, Anu Kantele

**Affiliations:** 1 Department of Bacteriology and Immunology, University of Helsinki, Helsinki, Finland; 2 Department of Medicine, University of Helsinki, Helsinki, Finland; 3 Division of Otorhinolaryngology, Department of Medicine, University of Helsinki and Helsinki University Hospital, Helsinki, Finland; 4 Helsinki University Hospital Laboratory (HUSLAB), University of Helsinki and Helsinki University Hospital, Helsinki, Finland; 5 Occupational Health and Environmental Medicine, Department of Public Health, University of Turku, Turku, Finland; 6 Inflammation Center, Clinic of Infectious Diseases, University of Helsinki and Helsinki University Hospital, Helsinki, Finland; 7 Unit of Infectious Diseases, Department of Medicine/Solna, Karolinska Institutet, Stockholm, Sweden; Monash University, AUSTRALIA

## Abstract

**Background:**

Despite the high frequency of upper respiratory tract (URT) infections and use of the nasal mucosa as route for vaccination, the local immune mechanism and dissemination of effector lymphocytes from the URT have been insufficiently characterized. To devise a single-cell approach for studying the mucosal immune response in the URT, we explored URT-originating B effector lymphocytes in the circulation of patients with one of two common respiratory infections, acute sinusitis or tonsillitis.

**Methods:**

Patients with acute sinusitis (n = 13) or tonsillitis (n = 11) were investigated by ELISPOT for circulating pathogen-specific antibody-secreting cells (ASCs) of IgA, IgG and IgM isotypes approximately one week after the onset of symptoms. These cells’ potential to home into tissues was explored by assessing their expression of tissue-specific homing receptors α_4_β_7_, L-selectin, and cutaneous lymphocyte antigen (CLA).

**Results:**

Pathogen-specific ASCs were detected in the circulation of all patients, with a geometric mean of 115 (95% CI 46–282) /10^6^ PBMC in sinusitis, and 48 (27–88) in tonsillitis. These responses were mainly dominated by IgG. In sinusitis α_4_β_7_ integrin was expressed by 24% of the ASCs, L-selectin by 82%, and CLA by 21%. The proportions for tonsillitis were 15%, 80%, and 23%, respectively. Healthy individuals had no ASCs.

**Conclusions:**

URT infections–acute sinusitis and tonsillitis–both elicited a response of circulating pathogen-specific plasmablasts. The magnitude of the response was greater in sinusitis than tonsillitis, but the homing receptor profiles were similar. Human nasopharynx-associated lymphoid structures were found to disseminate immune effector cells with a distinct homing profile.

## Introduction

The human upper respiratory tract (URT) is repeatedly exposed to a large variety of inhaled microbes; accordingly, upper respiratory tract infections (URTI) constitute the most common reason for emergency room visits in primary health care [1, 2]. High infection incidences, influenza epidemics, and increasing antimicrobial resistance among URT pathogens, all point to a need for more efficient vaccination strategies to protect this entry portal. Despite this, the local immune mechanisms defending the respiratory tract against pathogens have been incompletely characterized. To understand the immunity elicited in the URT, more needs to be learned about the targeting of the immune response from this site, i.e. the migration of nasopharynx-originating immune effector cells in the body.

Mucosal antibodies are considered elementary in the mucosal defense of the URT, as they interfere with the initial steps of infection, preventing a pathogen’s attachment, subsequent spread and invasion [3, 4]. Antigen encounter in a mucosa-associated lymphoid tissue activates antigen-specific lymphocytes which migrate to local lymph nodes and return via lymphatics and blood to various mucosal surfaces, primarily the original site, to carry out their effector functions [5, 6]. The plasmablasts only appear transiently in the circulation [7–11]. Indeed, in studies applying the right timing, circulating pathogen-specific plasmablast have been detected in upper respiratory infections caused by respiratory viruses [12] as well as after intranasal vaccination [13–15].

The dissemination of the newly activated plasmablasts or ASCs, a tightly regulated multi-step homing process, exhibits a significant degree of tissue selectivity [5, 6], where homing receptors (HR) and chemokine receptors (CCR) serve a central function. Activated ASCs express specific HRs and CCRs that recognize their specific ligands, addressins and chemokines, in the effector tissues [5, 6]. Consequently, a restricted cell population homes to specific tissues at a specific time. Tissue-specific HRs haven been identified: α_4_β_7_ integrin guides cells to intestinal mucosa [16], L-selectin to peripheral lymph nodes [17], and cutaneous lymphocyte antigen (CLA) to skin tissues [18].

It is worthy of note that the site of antigen encounter affects the HR repertoire on activating cells, and thus also the targeting of the specific immune response [6, 19–26].

As the localization of the effector lymphocytes can be influenced by the choice of immunization route, each potential inductive site deserves thorough investigation. It is not fully known which HRs guide cells to the respiratory tract, and the targeting of the response elicited at this site has remained inadequately studied in humans. We set up a single-cell study to explore the URT as an inductive site in natural bacterial infection. We characterized pathogen-specific ASC response in patients with acute sinusitis or acute tonsillitis, focusing on HR expression on ASCs and their trafficking potentials in the body.

## Materials and Methods

### Study design

**Fig 1:**
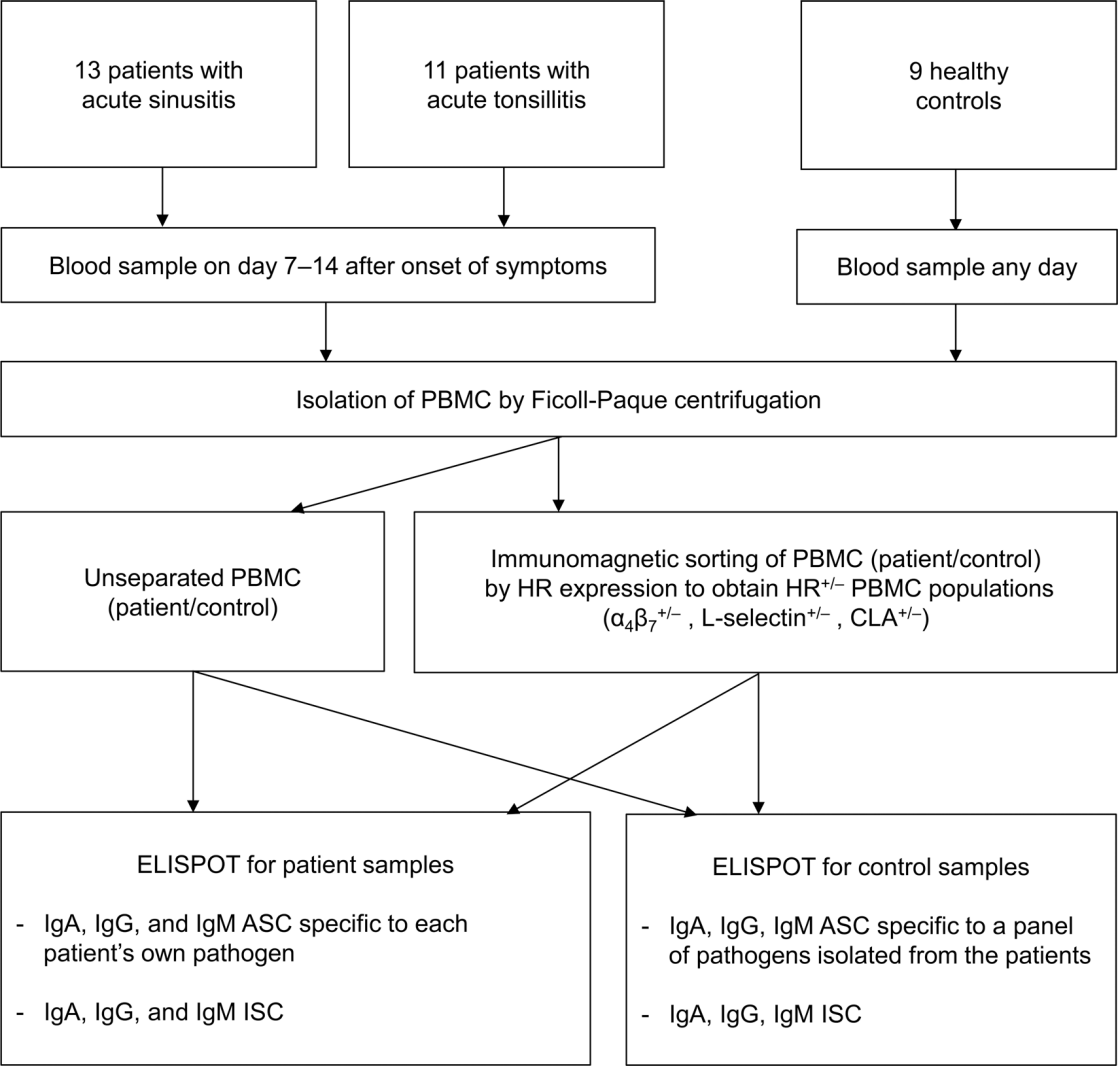
Flow diagram of the study.

We looked for pathogen-specific circulating ASCs in patients with acute sinusitis or tonsillitis. Pathogen-specific ASCs and all immunoglobulin-secreting cells (ISCs) and their isotype distributions were analyzed using enzyme-linked immunospot (ELISPOT) assay, and the expressions of HRs (α_4_β_7_–integrin, L-selectin, and CLA) were examined by combining immunomagnetic cell sorting and ELISPOT (Fig 1). While the ASCs represent a population of close to end-stage B cells producing antibodies specific to each patient’s own pathogen, all ISC denote the total of various ASC specific to the variety of recently encountered antigens.

The study protocol was approved by the Ethics Committee of the Helsinki University Hospital (411/E5/02:387/2002, approval for continuation 8122010). Healthy volunteers served as controls. Written informed consent was obtained from all volunteers.

### Patients and healthy volunteers

A total of 24 outpatients visiting the emergency room of the Department of Otorhinolaryngology at Helsinki University Hospital or the Central Hospital of Central Finland participated in the study, 13 with acute bacterial maxillary sinusitis and 11 with acute tonsillitis. The diagnosis of acute sinusitis was based on a combination of typical patient history (e.g. nasal congestion, purulent nasal discharge, facial pain or pressure, and impaired sense of smell) and purulent discharge with bacteria (≥10^5^ cfu / mL) obtained by maxillary irrigation. Likewise, the diagnosis of acute tonsillitis relied on typical patient history (e.g. fever, sore throat, anterior cervical lymphadenopathy) and *Streptococcus haemolyticus* growth in throat culture (≥10^5^ cfu / mL). We only enrolled patients with a positive bacterial culture and symptoms lasting less than two weeks. Table 1 summarizes the patients’ background data. Nine healthy volunteers, six women and three men, aged 29 to 46, served as controls.

**Table 1:** Patient demographics, clinical picture and number of specific ASCs.

	Sample no.	Age	Gender	Pathogen	Day^a^	Fever °C	CRP mg/L	ASCs^b^/10^6^ PBMC
**Sinusitis**	**S1**	53	F	*H*. *influenzae*	11	39	NA^c^	58
	**S2**	34	M	*S*. *pneumoniae*	10	39	45	268
	**S3**	19	M	*S*. *pneumoniae*	7	38.8	139	651
	**S4**	77	F	*H*. *influenzae*	10	NA	44	102
	**S5**	35	M	*S*. *pneumoniae*	10	37	NA	1256
	**S6**	31	M	*H*. *influenzae*	10	39	150	14
	**S7**	55	M	*S*. *anginosus*	9	<37	NA	10
	**S8**	24	F	*S*. *pneumoniae*	14	NA	119	104
	**S9**	25	F	*S*. *pneumoniae*	12	38	121	22
	**S10**	59	M	*H*. *influenzae*	7	<37	102	105
	**S11**	56	F	*S*. *pneumoniae*	7	<37	NA	205
	**S12**	35	F	*H*. *influenzae*	10	38	NA	229
	**S13**	23	M	*H*. *influenzae*	12	39	92	287
**Tonsillitis**	**T1**	33	F	*S*. *pyogenes*	7	NA	NA	84
	**T2**	35	F	*S*. *pyogenes*	7	NA	NA	171
	**T3**	29	F	*S*. *pyogenes*	7	39	NA	40
	**T4**	20	M	*S*. *pyogenes*	5	NA	57	11
	**T5**	31	M	*S*. *pyogenes /S*. *aureus*	7	41	140	8/11
	**T6**	25	F	*S*. *pyogenes*	7	39	240	51
	**T7**	23	M	*S*. *pyogenes*	9	39	176	24
	**T8**	28	F	beta-hemolytic *Streptococcus*	7	NA	57	55
	**T9**	25	F	beta-hemolytic *Streptococcus*	10	39	160	108
	**T10**	26	F	*S*. *pyogenes*	7	NA	NA	322
	**T11**	40	F	*S*. *pyogenes*	7	NA	NA	26

^a^ Symptoms in days indicate the number of days the symptoms had lasted before the blood sample was drawn.

^b^ (IgA + IgG + IgM) ASCs

^c^ NA = data not available.

### Bacterial pathogen

Maxillary irrigation (sinusitis group) was carried out and throat swabs taken (tonsillitis group) by an otologist during the first visit. The pathogen was isolated according to the routines of the microbiological laboratories of the attending hospitals. Each isolate was then suspended in 0.9% saline solution and killed by adding formaldehyde at a concentration of 2%. The concentration was adjusted to 10^8^ bacteria/ml PBS according to the McFarland standard [21] and the suspension used in the ELISPOT for pathogen-specific ASC (see below).

### Blood samples

Blood samples were drawn 5–7 days after the maxillary irrigation or throat culture. For sinusitis patients this was a routinely scheduled follow-up visit. To allow comparisons between the two groups, the same timing was used for tonsillitis patients. Table 1 presents time points of sampling with respect to the onset of symptoms.

### Isolation of mononuclear cells

Peripheral blood mononuclear cells (PBMC) were isolated from heparinized venous blood by Ficoll-Paque centrifugation as previously described [8].

### Separation of cells into HR-negative and -positive cell populations

PBMC were sorted into HR-positive and -negative cell subpopulations by their expressions of α_4_β_7_, L-selectin and CLA using immunomagnetic cell sorting as described earlier [20, 21]. In short, 3 x 10^6^ PBMC were incubated with one of the receptor-specific anti-human monoclonal antibodies (mAb): anti-L-selectin (Leu-8), anti-α_4_β_7_ (ACT-1) or anti-CLA (HECA-452, a gift from Dr S. Jalkanen and Dr E.C. Butcher). After washing, the cells were incubated with Dynal M-450 magnetic beads coated with sheep anti-mouse IgG (Oslo, Norway). After separation, the receptor-positive and receptor-negative subpopulations were analyzed for antibody production using the ELISPOT assay. We have previously reported on the efficiency of the separation process [20].

### Assay of circulating ASCs and ISCs

The ELISPOT analysis of pathogen-specific ASCs and all ISCs (IgA, IgG, and IgM) in the total PBMC population and the receptor-positive and -negative subpopulations was carried out as described previously [8, 9, 27, 28]. In brief, to assess pathogen-specific ASC, 96-well microtiter plates were coated with a suspension of each patient’s own pathogenic strain. In the ISC assay, human IgA, IgM, (Dako, Glostrup, Denmark) or IgG (Sigma, Immuno Chemicals, St. Louis, MO, US) -specific antisera (diluted in PBS) were used. After washing and masking the wells, aliquots of the total PBMC populations or the receptor-positive and negative subpopulations were added. The antibodies/ immunoglobulins secreted by the cells were then detected with alkaline phosphatase-conjugated anti-human IgA, IgG (Sigma) or IgM (Southern Biotech, Birmingham, AL) followed by the substrate (5-bromo-4-chloro-3-indolyl phosphate, Sigma) in melted agarose. Under a microscope, each spot was interpreted as a print of a single ASC or ISC.

The nine healthy volunteers, six women and three men, were examined for ASCs against a panel of four *Streptococcus pyogenes*, two *Haemophilus influenzae*, one *Staphylococcus aureus*, and one *Streptococcus pneumoniae* strain, each initially isolated from one of the patients in the present study.

### Statistical analysis

The frequencies of pathogen-specific ASCs (IgA+IgG+IgM)/10^6^ PBMC or all ISCs (IgA+IgG+IgM)/10^6^ PBMC were given as geometric means, and 95% confidence intervals (95% CI) counted using bootstrapping. The frequencies between the study groups were compared by independent-samples Kruskal-Wallis test (SPSS 22.01; SPSS Inc.) or independent-samples Median test in case of high variance across the outcomes [29].

For each patient, the percentages of ASCs expressing a given HR were calculated as follows: *% receptor-positive ASC = 100 x [number of ASC in receptor-positive population] / [total of ASC in receptor-positive and -negative populations]*. And respectively for ISCs of each patient or control: *% receptor-positive ISC = 100 x* [*number of ISC in receptor-positive population] / [total of ISC in receptor-positive and -negative populations]*.

The proportion of cells expressing the various HRs was given as arithmetic means ±SD, and, when appropriate, compared with independent-samples Mann-Whitney U test or related-samples Wilcoxon Signed Rank test. To obtain reliable statistics, we included only study subjects with ≥ 20 spots identified in the HR analyses. In all tests, p < .05 was considered significant.

## Results

### Number of ASCs and ISCs

ASCs specific to each individual’s own pathogen were detected in all patients with acute sinusitis (13/13) and acute tonsillitis (11/11) (Table 1). In acute sinusitis, the geometric mean of the pathogen-specific ASCs (IgA + IgG + IgM) /10^6^ PBMC was 115 (95%CI 46–282), and in acute tonsillitis 48 (27–88) (Fig 1). The response proved slightly higher in sinusitis than tonsillitis, p = .041. None of the six healthy volunteers had ASCs specific to any of the pathogens in the panel (see above).

**Fig 2:**
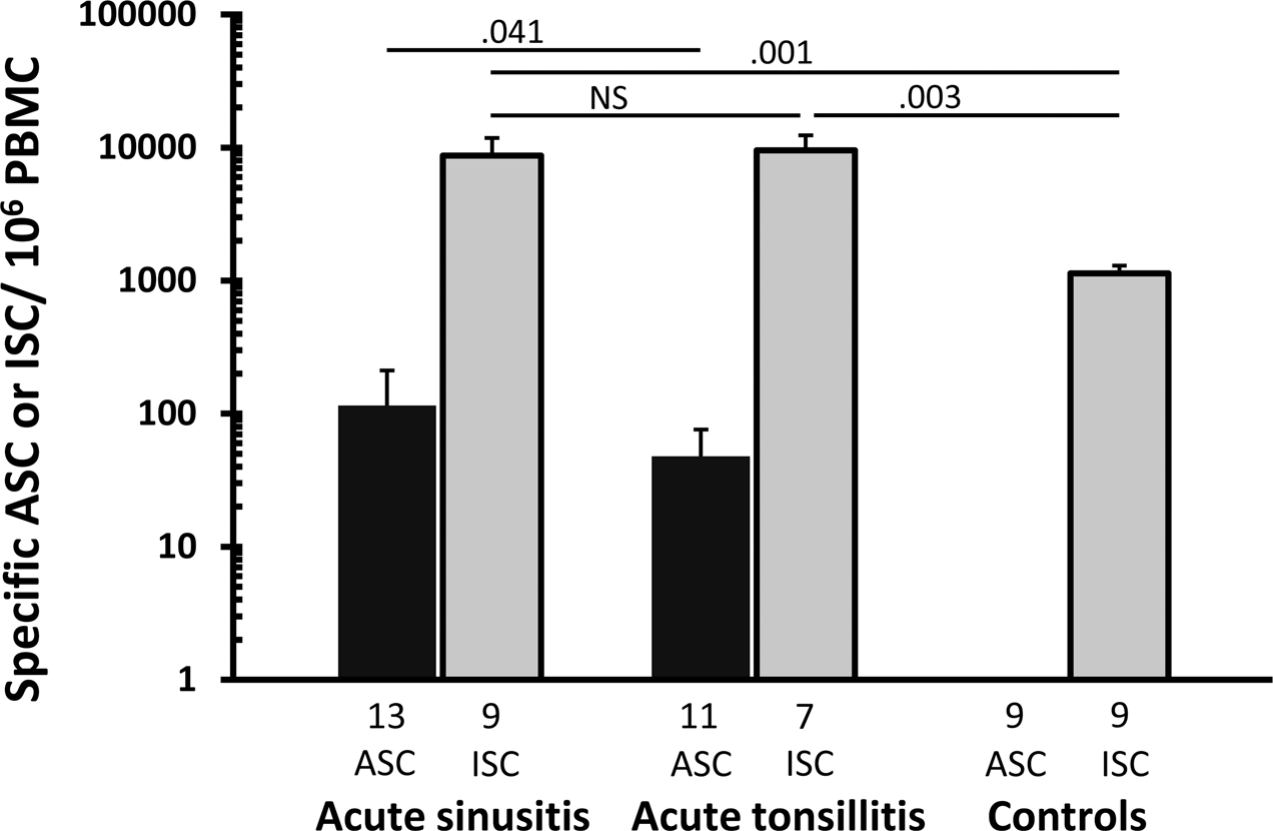
Geometric mean of pathogen-specific ASCs and all ISCs. The bars indicate geometric means (±SEM) of the number of ASCs and ISCs in the peripheral blood of patients with bacterial acute sinusitis, bacterial acute tonsillitis, and in healthy controls (IgA + IgG + IgM) ASCs or -ISCs/10^6^ PBMC). The patient samples were examined 7–14 days after the onset of symptoms. The number of volunteers from whom the data were pooled is shown under each bar. Differences between the distributions of the groups were tested with independent samples Median test (for ASC) or independent samples Kurskal-Wallis (for ISC). NS–p > .05.

The geometric mean of ISCs (IgA + IgG + IgM) /10^6^ PBMC in those with acute sinusitis was 8689 (95%CI 3952–17020), those with acute tonsillitis 9532 (5695–16554), and in healthy controls 1135 (887–1420) (Fig 2). The ISC number proved higher in the patient groups than controls (p = .001 for sinusitis and .003 for tonsillitis); no difference was seen between the two patient groups (p = .1).

### Ig isotype distributions of ASC and ISC

**Fig 3:**
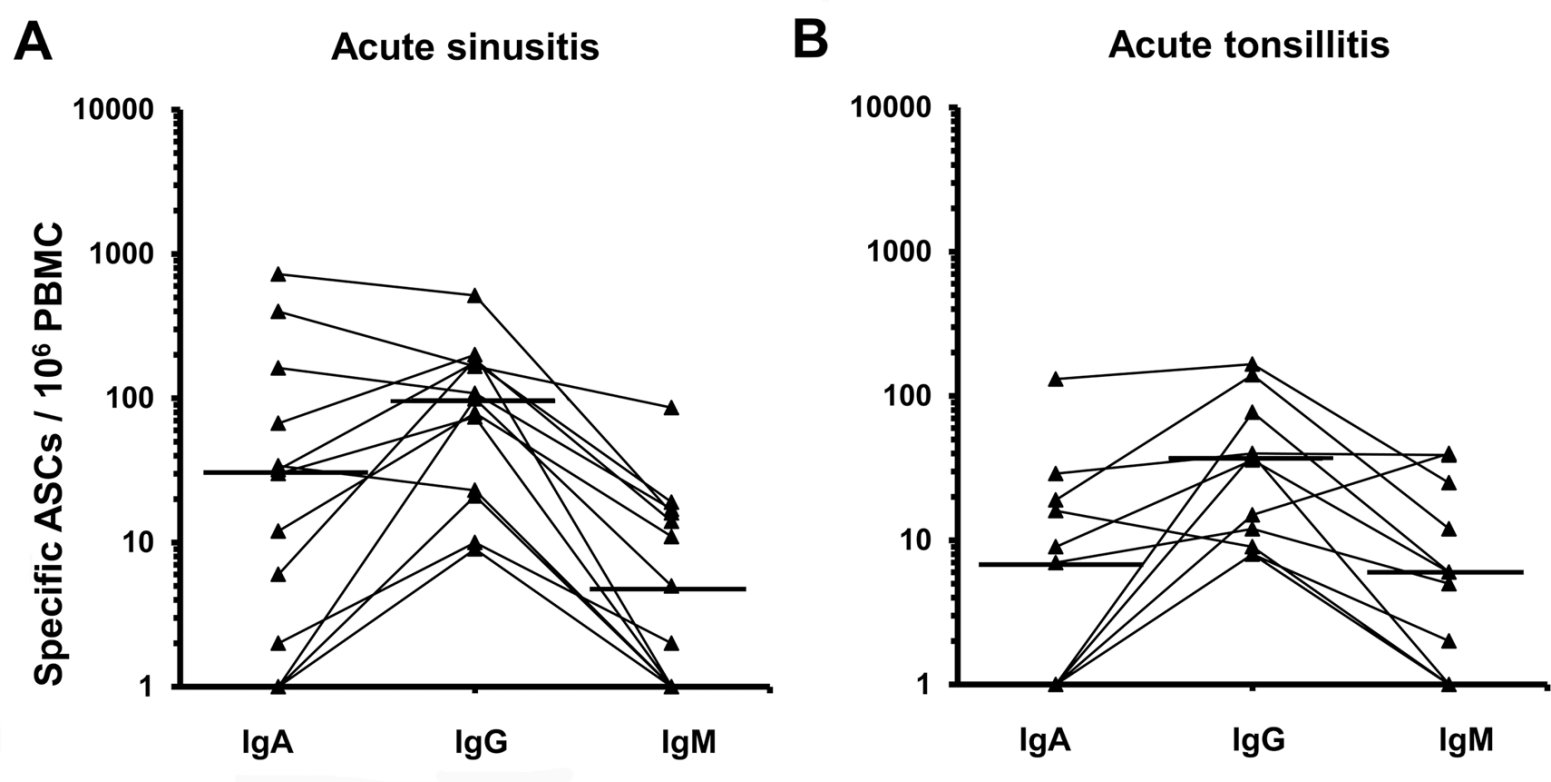
Isotype distributions of pathogen-specific ASCs in patients with (A) acute sinusitis and (B) tonsillitis. The dots represent the number of circulating pathogen-specific IgA, IgG or IgM ASCs (/10^6^ PBMC) of single patients, the values for different Ig-isotypes are joined with a line. The horizontal lines indicate geometric means (sinusitis n = 11, tonsillitis n = 7).

In the sinusitis group, the geometric mean was 72 (95% CI 32–142) /10^6^ PBMC for the pathogen-specific IgG-ASCs, 18 (4–77) for IgA-ASCs, and 5 (2–12) for IgM-ASCs (Fig 3A). IgG-ASCs were detected in all of these patients, IgA-ASC in 10/13, and IgM in 8/13; IgG isotype predominated in 9/13 and IgA in 4/13.

In the tonsillitis group, the geometric means of IgG-, IgA- and, IgM-ASCs were 29 (95%CI 14–60), 5 (2–15), and 6 (2–16) /10^6^ PBMC, respectively (Fig 3B). IgG-ASCs were detected in all patients, IgA-ASC in 6/11, and IgM in 8/11; IgG-ASCs predominated in 9/11 and IgA- or IgM-ASC each in 1/11 volunteers (Fig 3B).

Circulating ISCs (Fig 2) were seen in all three isotypes in all patients and controls (data not shown), indicating that none had agammaglobulinemia. IgG-ISC predominated in all three groups, IgA-ISC in one patient in both of the patient groups and in two control subjects.

### Expression of α_4_β_7_, L-selectin, and CLA on pathogen-specific ASCs and ISCs

**Fig 4:**
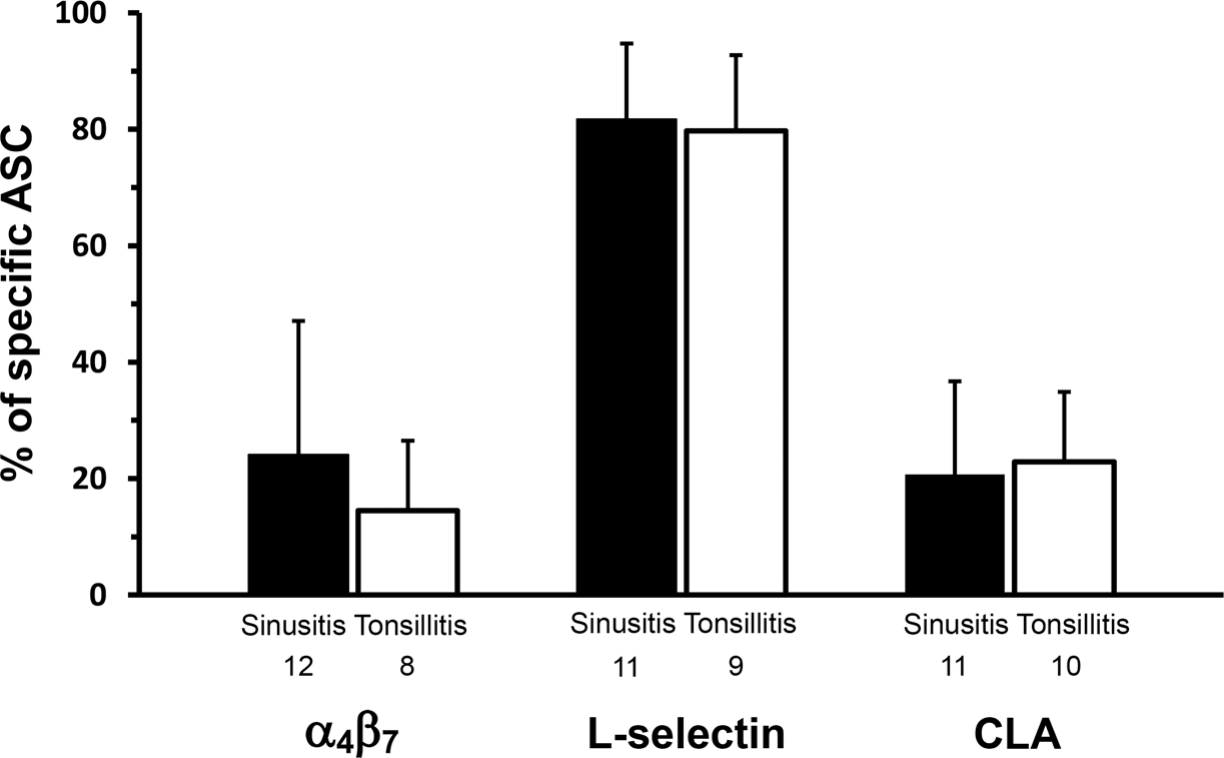
Expression of α_4_β_7_, L-selectin, and CLA on pathogen-specific ASCs in acute sinusitis and tonsillitis. The bars indicate arithmetic means (±SD) of percentages of cells expressing the given HR among all ASC. The number of patients from whom the data were pooled is indicated under each bar. No significant differences were seen between the study groups in the expressions of the various HR when tested using independent-samples Mann-Whitney U test.

The proportion of ASCs expressing the various HRs was similar in the two patient groups: α_4_β_7_ was expressed by 24% ± 23 of ASC in sinusitis and 15% ± 12 in tonsillitis (Fig 4). The respective figures for L-selectin were 82% ± 13 and 80% ± 13, and for CLA 21% ± 16 and 23% ± 12 (Fig 4). As for ISCs, α_4_β_7_ integrin was expressed by 35% ± 14, 38% ± 13 and 50% ± 13 of all ISCs in the sinusitis, tonsillitis and control groups respectively. The respective proportions for L-selectin were 65% ± 14, 73% ± 13, and 59% ± 9, and for CLA 27% ± 17, 31% ± 11, and 23% ± 10.

### Comparison of HR expressions between patients’ ASCs and ISCs

**Fig 5:**
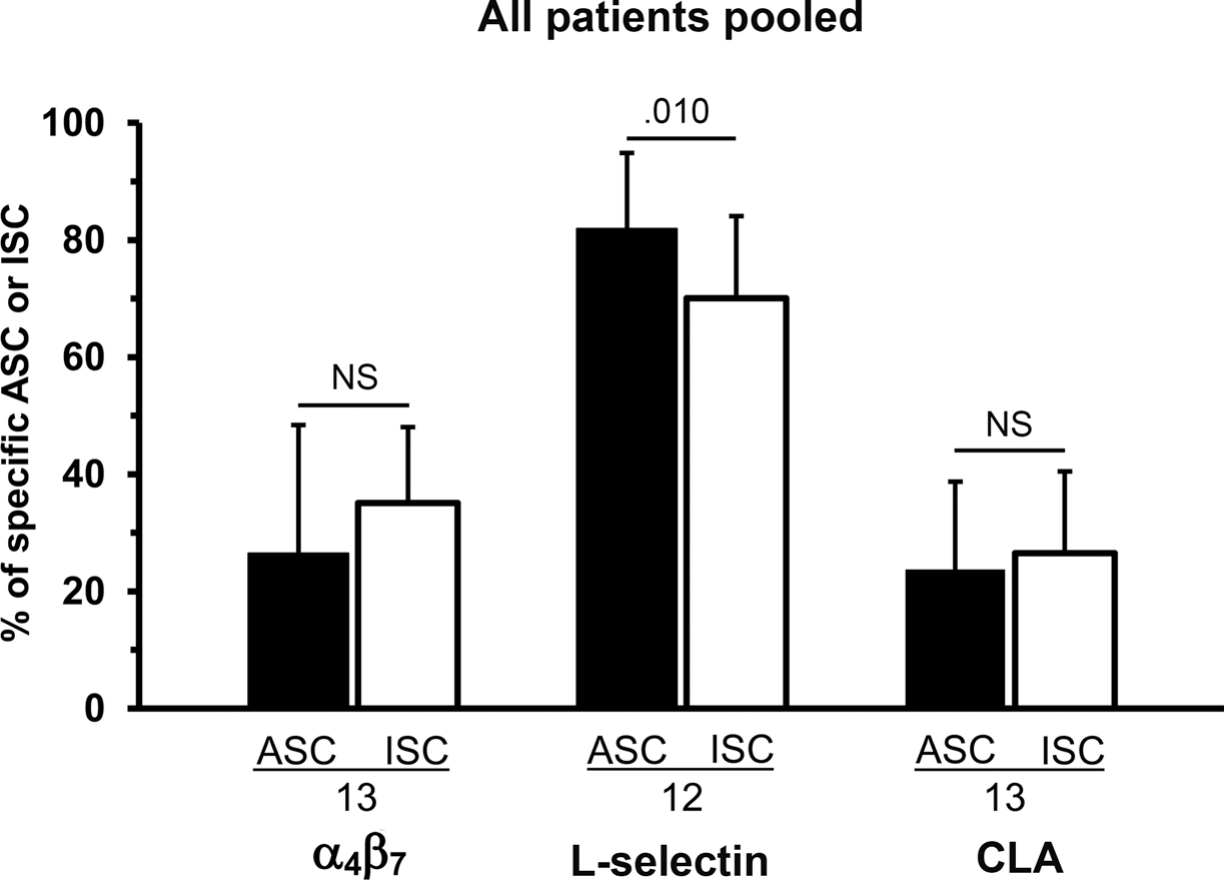
Comparison of proportions of α4β7-, L-selectin-, and CLA-expressing cells between ASCs and ISCs of the same patients. Data of patients with acute sinusitis and tonsillitis were pooled. The number of subjects is shown under each bar. Related-samples Wilcoxon Signed Rank test was used in comparisons between the same patients’ ASCs and ISCs; p-values are shown above the bars (NS = p > .05).

The HR data on the two patient groups’ ASCs were pooled to allow comparison between the same patients’ ASCs and ISCs. The proportion of L-selectin^+^ cells was higher among ASCs than ISCs (p = .010), while that of α_4_β_7_ or CLA did not differ between ASCs and ISCs (Fig 5).

## Discussion

This study is the first to show that acute bacterial URTI is followed by a response of circulating pathogen-specific plasmablasts, and that human nasopharynx-associated lymphoid structures disseminate these immune effector cells with a unique homing profile.

### Response of URT-originating plasmablasts–comparison with other mucosal sites

Circulating pathogen-specific plasmablasts were found in all patients with acute sinusitis or tonsillitis, concurring with studies showing respective cells in viral URTI [12] and after intranasal immunization [13–15, 22, 23]. Likewise, we recently identified circulating pathogen-specific plasmablasts in patients with LRTI caused by *Streptococcus pneumoniae* [28]. Such circulating mucosa-originating plasmablasts representing cells newly activated at local inductive sites have also been found in infections at other mucosal sites, e.g. gastroenteritis [9, 21, 30] and lower [27, 31] and upper [27, 32, 33] urinary tract infection, both in adults [27, 31, 32] and children [33]. In the URTI the inductive site is the nasopharynx-associated lymphoid tissue, NALT, which consists of Waldeyer’s ring (refers mainly to adenoids and palatine tonsils) [34] and occasional lymphoid follicles scattered in the nasal mucosa [34, 35]. The appearance of such NALT-originating lymphocytes in the circulation may offer a valuable tool for research into the URT mucosal immune system in humans.

### Timing of blood sampling

Plasmablasts are known to appear only transiently in the circulation. The kinetics of this response has been explored with oral [7, 8, 10] and rectal [19] vaccines, and in gastroenteritis [11]. The cells appear in the circulation approximately three days after antigen encounter, peak on day seven, and disappear by day 14–16 [7–11, 36], the latter a consequence of their homing to the effector sites. A persisting antigenic stimulus may prolong the presence of plasmablasts in the blood [10, 11]. Accordingly, to catch the peak or come near it, we drew the blood samples on day 7–14 after the onset of symptoms. This timing appeared feasible, since plasmablasts were detected in all subjects. As we enrolled only patients with acute disease, the timing appears comparable in the two groups.

### Magnitude of plasmablast response

The magnitude of the URTI plasmablast response was found to be slightly lower than that found in our recent LRTI study [37]. This may reflect the less severe nature of sinusitis or tonsillitis compared to pneumonia, analogous to lower *vs* higher urinary tract infection reported in our previous study [27]. In sinusitis the plasmablast response was somewhat greater than in tonsillitis, which may imply a wider dissemination to local inductive sites in sinusitis.

### Immunoglobulin distribution of plasmablast response

IgG proved the most prevalent Ig isotype in both sinusitis and tonsillitis. This accords with previous vaccination studies of the human URT: tonsillar cholera immunizations [13] and intranasal influenza vaccinations [15] have been shown to induce an IgG-dominated plasmablast response in the circulation [13, 15] and in tonsils and adenoids [13]. IgG isotype also dominates in the plasmablast response in pneumonia [28]. Indeed, despite the well-known role of IgA as the mucosal antibody at all other mucosal sites [7–9, 21, 27, 28, 38] IgG appears to serve a more important function in the respiratory tract. Interestingly, besides the predominance of IgG plasmablasts in both, IgA cell response was seen more commonly in patients with sinusitis (10/13) than tonsillitis (6/11). The presence of a mucosal secretory system with abundant polymeric IgA, J-chain and specific epithelial elements of pIgR/SC has been reported in adenoids, but not in palatine tonsils [34]. Thus, the greater frequency of IgA-ASC responses in patients with sinusitis than tonsillitis may reflect a more pronounced B cell priming in adenoids in this disease.

### Homing profiles of plasmablasts in tonsillitis and sinusitis

The overall homing profiles of the pathogen-specific plasmablasts were similar in the two study groups: most cells expressed L-selectin, and less than one quarter α_4_β_7_ or CLA. This similarity suggests involvement of the same activation sites. The data reveal a distinct homing profile for plasmablasts elicited at the URT: the profile differs not only from that described for diarrhea (α_4_β_7_^high^, L-selectin^low^, CLA ^neglible^) [21, 32] or upper urinary tract infection (α_4_β_7_^moderate/high^, L-selectin^moderate/high^, CLA^low^) [32], but also for lower respiratory tract infection (α_4_β_7_^moderate^, L-selectin^high^, CLA^low^) [37]. The profile appeared to resemble most closely the systemic type of immune response (α_4_β_7_^low^, L-selectin^high^ CLA ^low^) elicited by parenteral vaccination [32, 20, 23].

As the site of antigen encounter determines the homing commitments of the newly activated lymphocytes [6, 19–26], it is of interest to consider each of the HR separately. The high proportion of L-selectin^+^ plasmablasts (sinusitis 80%, tonsillitis 82%) corresponds to that described for pneumonia (79%) [37] or elicited by nasal vaccination (80–90%) [23]. Consistently, the entire respiratory tract is characterized by an abundance of peripheral-node addressins (PNAd) [39, 40]. As for T cells, only a low/negligible frequency of L-selectin-expressing cells has been found in human lungs [41, 42], suggesting that strong L-selectin involvement in the respiratory tract may be unique for effector B cells. Moreover, comparison with infections at other mucosal sites [32, 21, 37] suggests that the great frequency of L-selectin^+^ cells may only be characteristic of URT- and LRT-originating plasmablasts. Likewise, in the present study, L-selectin was expressed more frequently by the pathogen-specific plasmablasts than the same patients’ total population of ISC, the latter comprising a collection of end-stage B cells recently activated at various sites of the body.

As regards α_4_β_7,_ the data do not suggest a central function for it in targeting URT-originating plasmablasts. Even though the main ligand for α_4_β_7_, the intestinal MadCAM-1, is reported to be expressed also on venules of the URT, only 24% (in sinusitis) and 15% (in tonsillitis) of the URT-originating plasmablasts expressed α_4_β_7_. This contrasts with our previous investigation of the LRT, where a substantial expression of α_4_β_7_ was seen in pneumonia patients (44%) [37]. Our data also contradict previous HR studies of URT-originating B cells induced by intranasal immunization; Quiding-Järbrink et al have reported considerable proportions of both α_4_β_7-_ and L-selectin-expressing cells among plasmablasts after intranasal cholera toxin immunization [23]. The reason for this discrepancy remains unclear. It could reflect the nature of the antigen (cholera toxin is an immunoadjuvant), for example, or alternatively, it could attest to differing inductive sites in intranasal vaccination vs natural infection. The various parts of Waldeyer’s Ring may equip immune effector cells differently, as suggested by the differences in the IgA system [34] discussed above. The response elicited by intranasal vaccination might thus be predominantly stimulated in the adenoids, and in both adenoids and palatine tonsils in natural infection. However, an acute infection can occasionally elicit a response with a great proportion of α_4_β_7_ cells, as evidenced by our two sinusitis cases (65% and 77%); this may suggest a more central function for adenoids as inductive sites in these patients.

The low frequency of CLA (21% in sinusitis and 23% in tonsillitis) apparently ensures the cells’ efficient targeting to the upper respiratory tract, avoiding a simultaneous “loss” of cells to undesired sites, such as the skin.

Our assay as such does not provide data on possible co-expression of the various HR. However, were the proportions of receptor-positive cells with various HR totaled (α_4_β_7_-, L-selectin- and CLA-positive cells), the result would exceed 100% in 9/13 and 6/11 patients with tonsillitis or sinusitis, respectively.

### Limitations

Two limitations deserve to be mentioned: the small number of subjects and coverage of markers. We only investigated three homing receptors, other potential HRs as well as chemokine receptors were excluded. However, we have applied the same selection in our previous research into the LRT, gastrointestinal and urinary tract infections, which enables comparison between the various mucosal sites with respect to each of these markers.

## Conclusions

We demonstrated that acute bacterial URTI induces pathogen-specific plasmablasts in the circulation, with IgG plasmablasts as the predominating isotype. A characteristic homing profile with a high proportion of L-selectin and scant α_4_β_7_ and CLA, was revealed for the upper airways, differing from that reported previously for infections at other mucosal sites.

## Abbreviations

URT: upper respiratory tract
ELISPOT: enzyme-linked immunospot assay
ASC: antibody-secreting cell
CLA: cutaneous lymphocyte antigen
URTI: upper respiratory tract infection
HR: homing receptor
ISC: immunoglobulin-secreting cell
PBMC: peripheral blood mononuclear cell
NALT: nasopharynx-associated lymphoid tissue
LRT: lower respiratory tract
LRTI: lower respiratory tract infection
